# Integration of Carbon Nanotubes into Manganese Dioxide Nanorods for Enhanced Enzymeless Electrochemical Glucose Sensing with High Sensitivity and Selectivity

**DOI:** 10.3390/bios15040215

**Published:** 2025-03-27

**Authors:** Khawtar Hasan Ahmed, Alonso Moreno Zuria, Mohamed Mohamedi

**Affiliations:** Institut National de la Recherche Scientifique (INRS), Énergie Matériaux Télécommunications (EMT), 1650, Boulevard Lionel-Boulet, Varennes, QC J3X 1P7, Canada

**Keywords:** manganese oxide nanorods, carbon nanotubes, hydrothermal, non-enzymatic sensor, glucose detection, interferences detection

## Abstract

Freestanding electrode designs, cost-effective catalysts, and enhanced electrical conductivity are crucial for improving the performance of fourth-generation non-enzymatic glucose electrochemical sensors. These factors enable more efficient, scalable, and durable sensors with better sensitivity, stability, and affordability for real-time glucose monitoring. In this study, we explore a freestanding electrode design combining carbon nanotubes (CNTs) with MnO_2_ nanorods to enhance charge transfer, increase surface area, and optimize catalytic activity. This CNTs/MnO_2_ electrode demonstrates exceptional catalytic activity for glucose oxidation, achieving a high sensitivity of 309.73 µA cm^−2^ mM^−1^ within a linear range of 0.5 to 10 mM—well above typical physiological glucose levels (3–8 mM), with a detection limit of 0.19 mM at a signal-to-noise ratio of 3. The electrode also shows excellent durability and remarkable selectivity for glucose over common interferents like ascorbic acid and uric acid, as well as antifouling properties in the presence of KCl. These attributes are essential for accurate glucose detection in complex biological samples. The integration of MnO_2_ nanorods with CNTs in freestanding nanostructures opens up exciting opportunities for developing high-performance, robust electrochemical sensors for diverse applications.

## 1. Introduction

Glucose is an essential source of energy for our bodies, but variations from normal levels can result in serious health complications. When glucose levels drop too low, a condition known as hypoglycemia (below 4 mmol/L) occurs, while excessively high levels are referred to as hyperglycemia (above 7 mmol/L) [1]. Diabetes is a metabolic condition where the body either fails to produce insulin (Type I diabetes) or cannot utilize it properly (Type II diabetes). This impairment hinders glucose from entering the cells, causing it to build up in the bloodstream, which may damage blood vessels. Proper measurement and monitoring of blood glucose levels are crucial for diagnosing and managing diabetes. Neglecting this can lead to severe complications such as cardiovascular diseases, heart attacks, arthritis, strokes, vision impairment, kidney failure, nerve damage, and amputations due to gangrene [2]. Given the potential for serious outcomes associated with uncontrolled blood glucose, creating reliable and affordable glucose monitoring devices remains a significant challenge.

Ongoing research is focused on developing fourth-generation electrochemical sensors. These advanced technologies seek to overcome the limitations of previous generations, improving performance, accuracy, and the overall user experience. Typically, these sensors utilize advanced materials and features to improve the effectiveness of glucose monitoring. Fourth-generation glucose sensors are characterized by improved selectivity, continuous monitoring capabilities, miniaturization, wearability, wireless connectivity, enhanced biocompatibility, increased longevity, and intuitive user interfaces [3,4]. Although previous enzymatic glucose sensors (EGS) have been essential for glucose level monitoring, they do not qualify as fourth-generation technologies. Recent developments in this field may involve non-enzymatic glucose sensors (NEGS), implantable devices, microfluidic sensors, and other cutting-edge methods that broaden the potential for glucose sensing.

The most studied materials for electrochemical detection of glucose in NEGS include carbon-based materials such as carbon nanotubes, graphene, and graphene oxide [5], as well as metal nanoparticles like Au, Ag, and Pt [6], and conducting polymers [7]. Metal oxides are commonly investigated for NEGS due to their catalytic properties, conductivity, and stability. Key metal oxides include SnO_2_, ZnO, Fe_2_O_3_, Co_3_O_4_, NiO, MnO_2_, and RuO_2_ [8]. These metal oxides can be combined with various nanostructures and conductive polymers to enhance the electrochemical performance of glucose detection in NEGS. For further information, readers are referred to the comprehensive review by Hwang et al., which explores recent advancements in NEGS [9].

Among the several metal oxides, manganese dioxide (MnO_2_) often stands out as a strong contender for sustainable and effective glucose electrochemical sensors. Indeed, MnO_2_ is relatively abundant and widely available. MnO_2_ can be easily synthesized using various straightforward and adaptable methods, such as sol-gel processes, hydrothermal synthesis, and electrochemical deposition. These synthesis methods are often environmentally friendly, especially when using water-based or non-toxic precursors. Finally, MnO_2_ has shown good biocompatibility, particularly in nanostructured forms, making it suitable for biomedical applications. Nonetheless, the existing literature contains limited studies specifically examining MnO_2_-based glucose sensors [10,11,12,13].

In our previous study, we demonstrated that tetragonal α-MnO_2_ nanorods, synthesized on microfibrous carbon paper, exhibited good performance as non-enzymatic glucose sensing [14]. These freestanding (binderless) electrodes exhibited a high sensitivity of 143.82 µA cm^−2^ mM^−1^ in the range of 0.01 to 15 mM and a limit of detection (LOD) of 0.282 mM for glucose [14]. Notably, the MnO_2_ electrode demonstrated exceptional selectivity over ascorbic acid and uric acid. In contrast, the gold electrode exhibited a lower sensitivity of 52.48 µA cm^−2^ mM^−1^ within the 1 to 10 mM range. These results highlight the superior performance of MnO_2_ nanorods for glucose monitoring. In this work, we aim to investigate the synergistic effects of incorporating carbon nanotubes (CNTs) into MnO_2_ nanorods (NRs) to enhance their electrochemical performance. Specifically, we seek to improve the sensitivity and lower the detection limits for glucose by leveraging the high conductivity and large surface area of CNTs. We will also assess the selectivity of the CNTs–MnO_2_ composite electrode against common interferents such as ascorbic acid (AA), uric acid (UA), and fouling ionic compounds like KCl, with the goal of improving glucose detection accuracy. Additionally, we will evaluate the stability and longevity of the hybrid electrode compared to stand-alone MnO_2_ nanorods, with an emphasis on optimizing its electrochemical response for more reliable diagnostic applications.

## 2. Material and Methods

### 2.1. Reagents

NaOH (Fisher Scientific, Nepean, ON, Canada, purity +97%), Glucose (Sigma-Aldrich, Oakville, ON, Canada), ascorbic acid (Sigma, purity 99%) and uric acid (Sigma, purity 99%), HCl (Sigma-Aldrich, concentration 37%), KMnO_4_ (Fisher Scientific, purity 99%), KCl (Sigma-Aldrich, purity 99%) were used without further purification. Carbon paper (CP) (Toray carbon paper, TGP-H-60) was obtained from Fisher Scientific Canada, Ontario, Canada.

### 2.2. Synthesis of CNTs/MnO_2_ Freestanding Nanocomposite

The synthesis of CNTs directly on CP substrates was conducted using a chemical vapor deposition (CVD) method, following refined protocols developed in our lab [15]. Specifically, CNTs were produced on the CP at a temperature of 700 °C, employing nickel as a catalyst. To initiate the process, a thin nickel layer (approximately 5 nm) was applied to one side of the CP using the sputtering technique. Subsequently, CNTs were grown on the CP/Ni substrate through CVD, utilizing gas flows of acetylene as the carbon source, alongside hydrogen and argon as carrier gases, with respective flow rates set at 20, 100, and 140 sccm.

**Scheme 1:**
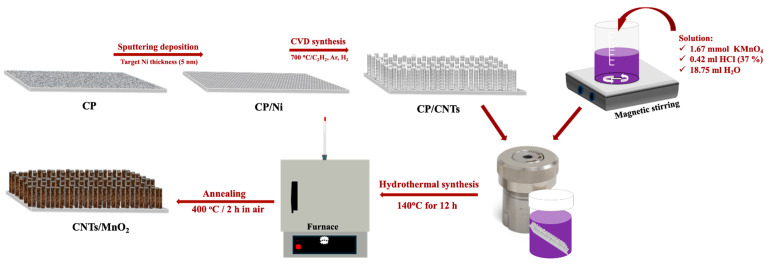
Procedure of fabrication of CNTs/MnO_2_-NRs.

For the synthesis of MnO_2_, we employed the hydrothermal method using KmnO_4_ as the precursor. Initially, 1.67 mmol of KMnO_4_ was dissolved in 18.75 mL of ultrapure deionized water (Millipore Milli-Q, resistivity 18.2 MΩ·cm, Sigma-Aldrich, Oakville, ON, Canada) and stirred for 15 min until fully dissolved. Afterward, 0.42 mL of concentrated HCl was added, and the mixture was stirred continuously for 2 min. Next, a piece of CP/CNTs was carefully placed into a 25 mL Teflon-lined stainless-steel autoclave. The KMnO_4_-HCl solution (prepared above) was then carefully added to the autoclave, ensuring the solution completely covered the CP/CNTs. The volume of the solution added is less than the total volume of the autoclave, which is 25 mL. The autoclave was securely sealed and subsequently heated in an oven at 140 °C for a duration of 12 h to facilitate the synthesis process. Upon completion of the reaction, the reactor was allowed to cool naturally to room temperature. The resultant CP/CNTs/MnO_2_ composites were then carefully washed with deionized water to remove any residual impurities. Finally, the samples were annealed in air at 400 °C for 2 h to improve their structural integrity and enhance the material properties (Scheme 1).

### 2.3. Materials Structural Characterization

The surface properties of the synthesized samples were analyzed using a TESCAN VEGA3 scanning electron microscope (SEM) (Brno, Czech Republic) set to 20.0 kV. Crystallinity was evaluated through X-ray diffraction (XRD) with a Bruker D8 Advance diffractometer, utilizing a Cu Kα source and operating at 40 kV and 40 mA. Diffraction data were gathered with a step size of 0.04° and an acquisition duration of 2 s per step. Additionally, Micro–Raman spectroscopy was employed to investigate the structural integrity of the samples. Raman spectra within the range of 100 to 2000 cm^−1^ were recorded with a Renishaw (inVia Reflex, Mississauga, ON, Canada) system, using a 532 nm laser at a low power setting of 0.1 mW (1% of 10 mW). The analyzed spot size was 2 μm, and for each sample, three spectra were collected, each taking 50 s to acquire, allowing for an averaged spectrum to be produced.

### 2.4. Electrochemical Measurements

To assess electrochemical performance, an electrochemical analyzer (Eco Chemie PGSTAT302 potentiostat/galvanostat, Metrohm Autolab, Utrecht, The Netherlands) was used. Measurements were performed at room temperature in a three-compartment electrochemical cell, which included a platinum coil as the counter electrode, an Ag/AgCl reference electrode in a 4 M KCl solution, and a rectangular CP/CNTs/MnO_2_ working electrode. To minimize the impact of ohmic drop, the reference electrode was positioned near the working electrode and separated from the electrolyte by a Luggin capillary. A 0.1 M NaOH aqueous solution, deoxygenated by bubbling argon for 20 to 30 min before each measurement, was used as the electrolyte.

For the medium-term stability test, glucose was added in successive increments, and the chronoamperogram was recorded after each addition. The measurement was continued until the steady-state current was reached. This process was repeated at each concentration to construct the calibration curve. The long-term stability test was conducted using chronoamperometry in the presence of 7 mM glucose, with continuous stirring at an applied potential of 0.18 V. The chronoamperogram was recorded over a period of 7 days (t = 604,800 s), with measurements taken at 70-s intervals, to monitor the sensor’s performance and assess its stability over time.

In this study, NaOH was chosen for its ability to enhance the electrochemical performance of non-enzymatic glucose sensors by optimizing the oxidation reaction of glucose at the electrode surface, thus improving sensitivity and reliability. This approach is commonly used in the sensor community, as NaOH provides an alkaline environment that facilitates the electrochemical processes involved.

## 3. Results and Discussion

### 3.1. Structural Characterization

**Figure 1:**
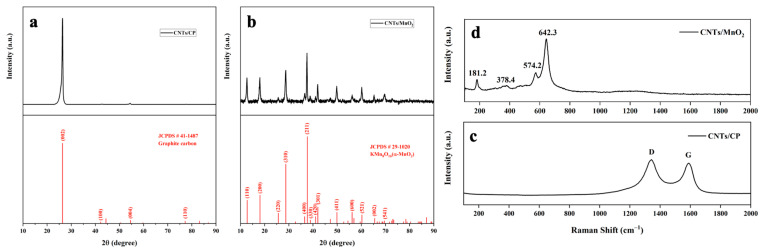
XRD patterns of (**a**) CP/CNTs and (**b**) CNTs/MnO_2_. Raman spectra of (**c**) CP/CNTs and (**d**) CNTs/MnO_2_.

XRD analysis was conducted to investigate the crystal structures of both the CNTs substrate and MnO_x_. As shown in Figure 1a, the CNTs substrate exhibits a prominent peak at 26.4° (2θ), along with three smaller peaks at 42.7°, 54.5° and 77.6° corresponding to the (002), (100), (004), and (110) planes of the graphite carbon substrates, respectively (JCPDS #41-1487). In Figure 1b, the XRD profile of the as-synthesized MnO_x_ onto CNTs reveals peaks that correspond closely to the planes of cryptomelane (KMn_8_O_16_), as documented in JCPDS #29-1020. This compound represents the typical tetragonal α-MnO_2_ phase within the manganese oxide family, characterized by the presence of K^+^ ions within the 2 × 2 tunnels. This structural arrangement not only stabilizes the crystalline framework of the α phase but also helps maintains charge balance within the material [16]. Figure 1c,d present the Raman spectra of both CNTs and CNTs/MnO_x_, respectively. The Raman spectrum of the CNTs substrate displays two prominent bands at approximately 1343.7 cm^−1^ and 1590.6 cm^−1^. These bands correspond to the D band, attributed to *A*_1g_ symmetry arising from edge or defect sites of carbon, and the G band, associated with *E*_2g_ symmetry indicative of sp^2^ carbon [17]. Figure 1d displays four distinct Raman peaks at 181.2, 378.4, 574.2, and 642.3 cm^−1^, all of which are characteristics of tetragonal α-MnO_2_ (also cryptomelane herein) [18]. It is important to note that the D and G band characteristics of the CNTs are not visible. This is likely attributed to the dense, thick layer of α-MnO_2_ that consistently covers the CNTs substrate.

**Figure 2:**
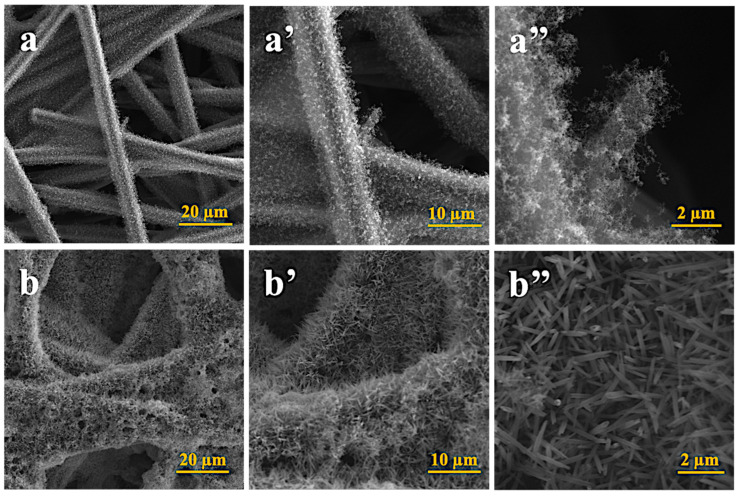
SEM images at increasing magnifications of CP/CNTs (**a**,**a’**,**a”**) and (**b**,**b’**,**b”**) of CNTs/MnO_2_–NRs.

**Figure 3:**
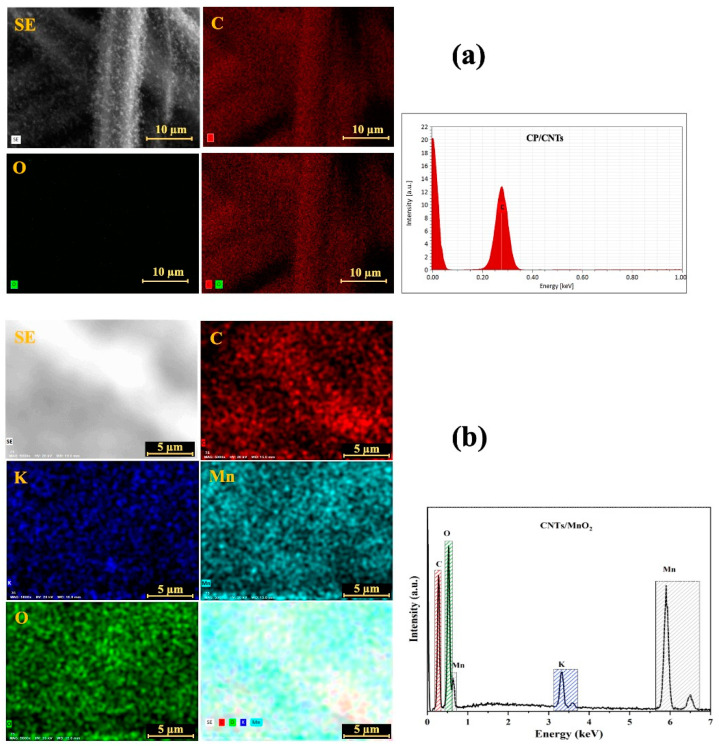
EDS mapping of C, K, Mn, and O elements of (**a**) CP/CNTs, and (**b**) CNTs/MnO_2_–NRs.

The SEM images of CP/CNTs, captured at progressively increasing magnifications in Figure 2a,a’,a”, clearly demonstrate that the CP substrate is densely coated with a rich layer of CNTs. Furthermore, an analysis of a broader area of the MnO_2_ layer subsequently grown on the CP/CNTs substrate, as shown in Figure 2b, reveals a thick and uniform deposit. The SEM images at higher resolutions of the deposit (Figure 2b’,b”) reveal a high density of MnO_2_ nanorods (NRs) arrays that are uniformly distributed. The Energy Dispersive X-ray Spectroscopy (EDS) mapping and spectra shown in Figure 3 provide detailed insight into the distribution of different elements across the sample. Figure 3a, corresponding to the CP/CNTs, reveals only dominant peaks for carbon, with no significant evidence of contamination. The EDS mapping further demonstrates a uniform dispersion of MnO_2_ onto the CNTs (Figure 3b). Additionally, the presence of potassium (K), as indicated by the EDS spectra in Figure 3b, suggests the formation of a KMn_8_O_16_ structure, which is consistent with the XRD data presented above.

### 3.2. Electrochemical Surface Area Measurements

**Figure 4:**
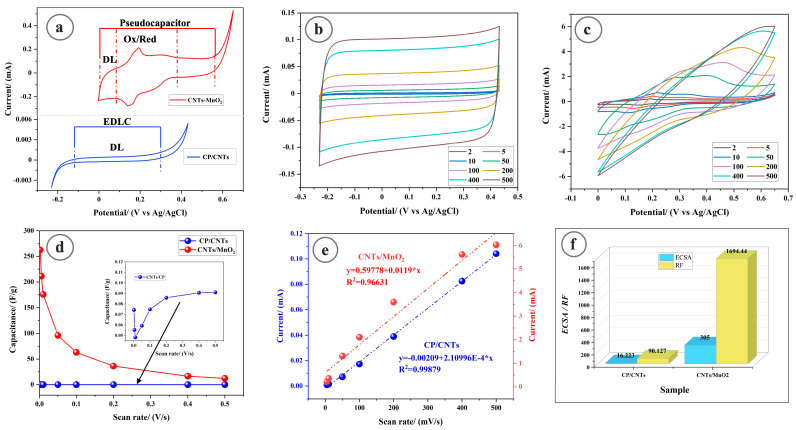
(**a**) Electrochemical windows of bare CP/CNTs and CNTs/MnO_2_–NRs in 0.1 M NaOH solution at a scan rate of 2 mV/s. (**b**,**c**) capacitive behavior of CP/CNTs and CNTs/MnO_2_–NRs, respectively. Numbers indicate the scan rate in mV/s. (**d**) Specific capacitance versus scan rate. (**e**) Non-faradic anodic currents extracted from (**b**,**c**) vs. scan rate. (**f**) Estimated *ECSA* and *RF* parameters.

Figure 4a compares the electrochemical windows for bare CNTs and CNTs/MnO_2_–NRs in a 0.1 M NaOH solution. Across the oxidation and reduction limits of NaOH, the cyclic voltammetry (CV) of bare CNTs shows stable currents, suggesting a pure double layer (DL) capacitance. In contrast, the CV of CNTs/MnO_2_–NRs displays symmetric peaks on both the anodic and cathodic sides in the 0.10 V and 0.24 V range, in addition to the DL region. The redox signals detected at the MnO_2_ electrode generally suggest the participation of surface Mn ions in redox processes. This observation highlights the pseudocapacitive characteristics of the charge storage process, where energy is stored via reversible redox reactions at the interface between the electrode and electrolyte [19]. In the CV of CNTs/MnO_2_ in NaOH solution, the observed redox peaks are attributed to the reversible redox reactions of Mn ions within the MnO_2_ structure. Specifically, these peaks correspond to the Mn^3+^/Mn^4+^ redox couple. During the reduction process, Mn^4+^ is reduced to Mn^3+^, while Mn^3+^ is oxidized back to Mn^4+^ during the anodic sweep. The Mn ions that participate in this redox reaction are typically the surface Mn^3+^ and Mn^4+^ ions, which are involved in the charge storage mechanism through reversible electron transfer at the electrode/electrolyte interface [20,21]. This pseudocapacitive behavior is a key feature of the MnO_2_ material, enhancing its electrochemical performance. The capacitive properties of both CNTs and CNTs/MnO_2_–NRs were further assessed through CV in a 0.1 M NaOH solution, using scan rates ranging from 2 to 500 mV/s. These results are shown in Figure 4b for CNTs and Figure 4c for CNTs/MnO_2_–NRs. The CV curves of the CNTs electrode display symmetrical rectangular shapes, typical of an electrochemical double layer capacitor (EDLC), where all charges are stored on the material’s surface. This capacitive behavior is observed across a potential window of 0.66 V for the CNTs electrode. The specific capacitance derived from the CV curves, is calculated using the formula *C_p_* = *Q*/(2 *m* × *v* × Δ*V*). In this equation, *Q* (A V) is the voltametric charge obtained by integrating the areas of both oxidation and reduction on the CV curve, *m* (g) is the mass of the active material on the working electrode, *v* is the scan rate (V/s), and Δ*V* (V) indicates the potential window of the CV. The *C_p_* values, plotted against the scan rate in Figure 4d, show that for the CNTs electrode, *C_p_* decreases as the scan rate increases, although a slight recovery is observed at higher scan rates. This kind of scan-rate dependence is quite common in materials like CNTs. The hydrophobic nature of as-made CNTs can significantly impact their electrochemical performance, particularly in terms of ion accessibility and wettability. Hydrophobic CNTs tend to have poor electrolyte interaction, limiting the ion diffusion into the CNTs structure, especially at lower scan rates. This could result in lower capacitance values due to ineffective electrolyte penetration. Activation, such as through CV, can help improve the wettability and surface chemistry of the CNTs, making them more hydrophilic and enhancing the ion diffusion during the charge-discharge process. As a result, activation can increase the electrochemical performance by allowing better electrolyte interaction, leading to higher capacitance. It is important to note that the *C_p_* value for the bare CNTs is relatively low, measuring less than 0.1 F/g. Likewise, the capacitive potential window of CNTs/MnO_2_–NRs is observed to be 0.65 V (Figure 4c). The rate performance of the CNTs/MnO_2_–NRs electrode, shown in Figure 4d, indicates that the *C_p_* decreases as the scan rate increases. A significant observation in Figure 4d is the high *C_p_* of CNTs/MnO_2_–NRs, attaining an exceptional value of 262.68 F/g at a scan rate of 2 mV/s. This value is over six times greater than the 42 F/g achieved by the CP/MnO_2_–NRs electrode [14]. This significant difference suggests that the CNTs-based structure enhances charge storage capacity due to factors such as improved conductivity, increased surface area, and greater electrochemical activity.

The electrochemical or electroactive surface area (*ECSA*) is a critical parameter in optimizing and understanding the performance of electrochemical systems, as it directly impacts the rates of electrochemical reactions. A larger *ECSA* indicates more active sites are available for reactions, enhancing the overall kinetics and efficiency of catalysts. The CV technique is one of the most popular techniques due to its simplicity and quick data acquisition, allowing for direct relationships between peak currents and *ECSA*. The electrochemical double-layer capacitance, *C_dl_* is calculated using the relationship *i_c_* = *v* × *C_dl_*, where *i_c_* signifies the double-layer charging current. Plotting *i_c_* against *v* yields a linear correlation, where the slope corresponds to *C_dl_*. Then, the *ECSA* is calculated using the *C_dl_*, expressed as *ECSA* = *C_dl_*/*C_s_*. Here, *C_s_* represents the standard specific electrochemical double-layer capacitance of the material, reflecting the capacitance of a smooth, planar surface per unit area under identical electrolyte conditions. However, developing perfectly smooth surfaces for each catalyst would improve the precision of *C_s_* measurements and the estimation of *ECSA*. However, this approach is often impractical due to the complexity of synthesizing certain catalysts, inherent material limitations that require rough or porous structures. It is notable that *C_s_* of 0.040 mF/cm^2^ has been reported for metal electrodes in 1 M NaOH solution [22]. For carbon materials, *C_s_* is roughly 13 µF/cm^2^, based on average values ranging from 5 to 20 µF/cm^2^ reported [23,24]. The roughness factor (*RF*) is determined by dividing the calculated *ECSA* by the electrode’s geometric area, which is 0.180 cm^2^. The *i_c_* values were extracted for CNTs and CNTs/MnO_2_-NRs within the non-faradic region in Figure 4b and Figure 4c, respectively. Figure 4e shows the *i_c_* values plotted against the scan rate. Remarkably, all curves follow a linear trend throughout the full range of scan rates measured. By analyzing the plot scan rates versus *i_c_*, the *C_dl_* values for the samples can be derived from the slopes of the graph. The measured *C_dl_* values are 0.21 mF for bare CNTs and 12.2 mF for CNTs/MnO_2_–NRs. The *C_dl_* of CNTs/MnO_2_–NRs is 2.8 higher than that of the CP/MnO_2_–NRs, which has a *C_dl_* of 4.3 mF [14]. Figure 4f presents the calculated *ECSA* and *RF* values for each sample. Notably, the *ECSA* of the CNTs is estimated at 16.22 cm^2^, while the CNTs/MnO_2_–NRs demonstrate an impressively large *ECSA* of 305 cm^2^ significantly higher than the 107.5 cm^2^ observed for the CP/MnO_2_–NRs system [14]. The incorporation of CNTs plays a crucial role in this enhancement, as their high conductivity and large surface area facilitate improved electron transfer and increased active sites for electrochemical reactions, thereby boosting the overall electrochemical performance of the hybrid material. This larger effective *ECSA* not only reflects the improved conductivity but also contributes to the higher specific capacitance observed in the CNTs/MnO_2_–NRs. A greater surface area allows for more ion adsorption and electron transfer during electrochemical processes, which can significantly enhance the performance of electrochemical sensing applications.

### 3.3. Voltammetric Detection of Glucose

**Figure 5:**
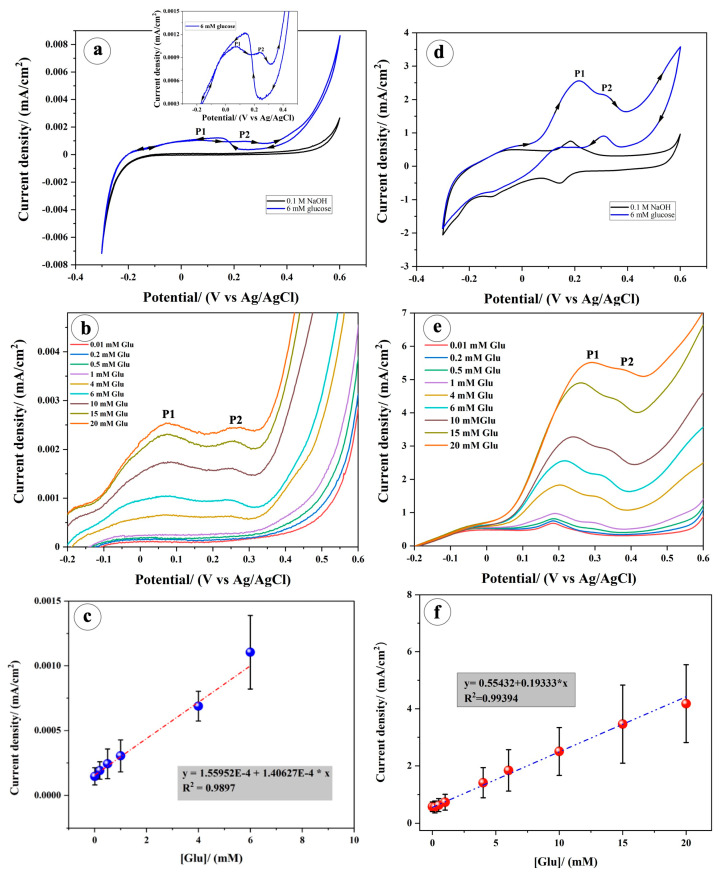
Electrochemical oxidation of Glu (**a**–**c**) on CNTs electrode, and (**d**–**f**) CNTs/MnO_2_–NRs electrode in 0.1 M NaOH solution recorded with a scan rate of 2 mV/s.

The enhanced *ECSA* and specific capacitance of CNTs/MnO_2_–NRs might significantly improve electrochemical sensing by increasing sensitivity through a greater number of active sites for analyte interaction, enabling the detection of lower concentrations. Their superior conductivity and ion transport may lead to faster response times, making the sensors more effective for real-time monitoring. Therefore, we undertook studies on glucose detection using these composites. Figure 5a shows the CV profile of the CNTs electrode with and without glucose (Glu). The addition of glucose reveals a noticeable activity for Glu oxidation, indicated by two weak anodic peaks, P1 and P2, at approximately 0.082 V and 0.234 V (Ag/AgCl), respectively. To achieve a quasi-steady state, slow linear scan voltammetry (LSV) measurements were conducted at a scan rate of 2 mV/s in a 0.1 M NaOH solution with various Glu concentrations, as illustrated in Figure 5b. It was found that the current peak densities for both P1 and P2 increased with higher Glu concentrations. The CNTs/MnO_2_–NRs electrode exhibited similar characteristics but displayed significantly higher current densities compared to the CNTs electrode (Figure 5d,e). The observed CV response aligns with the typical voltammetric pattern associated with Glu oxidation [25,26]. In the course of these investigations, peak P1 corresponds to the oxidation of glucose to gluconolactone, a reaction that involves two electrons. Conversely, peak P2 seems to be linked to the oxidation of gluconolactone to gluconic acid, which requires four electrons. The comparison between the CNTs and CNTs/MnO_2_–NRs electrodes reveals that the latter exhibits significantly higher current densities for Glu oxidation, indicating enhanced electrocatalytic activity.

Subsequently, the sensor’s sensitivity was assessed by examining the relationship between current peak density (P1) and Glu concentration. A linear regression analysis was conducted on the resulting data plot. The sensitivity is derived from the slope of the regression line, reflecting the change in current peak density with each unit increase in glucose concentration. To calculate the limit of detection (*LOD*), the formula *LOD* = 3.3 (*σ*/*s*) is applied, where *σ* denotes the standard deviation of the baseline signal, and *s* is the slope of the regression line. This equation estimates the minimum Glu concentration that the sensor can reliably identify, with the factor 3.3 ensuring an appropriate confidence level in the detection. Figure 5c illustrates the relationship between the maximum current peak, P1, and Glu concentration for the CNTs electrode. A strong linear correlation (R^2^ = 0.9897) is observed between 0.01 and 6 mM, which exceeds typical physiological glucose levels (3–8 mM). However, this electrode has a relatively low sensitivity of 0.140 µA cm^−2^ mM^−1^ and a *LOD* of 0.269 mM. In contrast, the CNTs/MnO_2_–NRs electrode shows an even stronger linear relationship (R^2^ = 0.9939) for peak P1 over a range of 0.01 to 20 mM, with significantly higher sensitivity of 193.33 µA cm^−2^ mM^−1^ and a lower *LOD* of 0.243 mM.

In summary, the reaction mechanism for glucose sensing remains unchanged with the presence of CNTs, as discussed in our previous work [14]. The addition of CNTs enhances the current density, thereby improving the sensitivity of the glucose sensor. CNTs provide a large surface area and excellent electrical conductivity, which facilitates efficient electron transfer during glucose oxidation. MnO_2_ acts as a catalyst for the glucose oxidation reaction, while CNTs enhance the overall conductivity and electron flow, resulting in a synergistic effect that improves the sensor’s performance.

### 3.4. Chronoamperometric Detection of Glucose

When studying sensing capabilities, the choice between CV and chronoamperometry (CA) hinges on the specific application requirements, the nature of the analytes, and the sample matrix. Researchers often use both techniques complementarily for optimal results. CV is advantageous for identifying multiple analytes with different redox potentials, but it has the drawback of continuous capacitive current, which can obscure signals from low-concentration analytes. This background noise complicates the detection of target species, especially at low concentrations. Even with background subtraction, completely eliminating the capacitive current from CV is not feasible. Any remaining trace of the capacitive current, even if low, may be comparable to the current associated with the target species, as the concentrations of analytes in sensors are often very small. In contrast, CA limits capacitive current effects primarily to the beginning of the potential step, allowing for a clearer measurement of the faradaic current as these effects diminish over time [27]. This makes CA particularly effective for detecting small amounts of electroactive species, providing a cleaner signal and enhancing sensitivity. Thus, CA is often the preferred choice for real-time monitoring and applications where sensitivity is critical.

**Figure 6:**
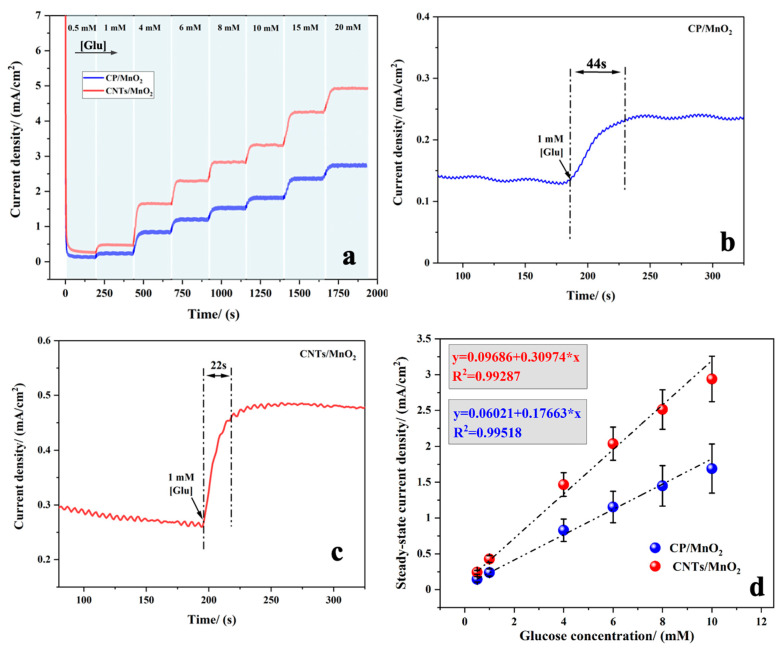
(**a**) Chronoamperometric (i–t) curve recorded for CP/MnO_2_–NRs and CNTs/MnO_2_–NRs electrodes for various concentrations of Glu (0.5–20 mM) in 0.1 M NaOH solution. Response time for glucose detection: (**b**) CP/MnO_2_–NRs electrode, and (**c**) CNTs/MnO_2_–NRs electrode. (**d**) Steady-state current density vs. [Glu] calibration plot.

In CA, the methodology for constructing a calibration curve involves applying a potential step to a working electrode while measuring the resulting current response over time. Once the potential is applied, the current is recorded as it evolves until it stabilizes at a steady-state value. This steady-state current (*i_ss_*), which reflects the maximum electron transfer rate at the electrode, is then plotted against the corresponding analyte concentrations. Figure 6a presents the CA response curves of CP/MnO_2_–NRs and CNTs/MnO_2_–NRs at a working voltage of 0.18 V vs. Ag/AgCl, showing the effects of successive additions of different Glu concentrations from 0.5 to 20 mM in a 0.1 M NaOH solution. We would like to clarify that the potential of 0.18 V was chosen for both CP/MnO_2_–NRs and CNTs/MnO_2_–NRs based on practical considerations. This potential is selected just before the peak oxidation potential for glucose oxidation. It is important to note that chronoamperometry cannot be conducted at the peak position because, at this point, glucose oxidation is already occurring at maximum efficiency, and no further glucose is available for oxidation. By choosing 0.18 V, we ensure that the measurement occurs in a region where glucose is still present and being oxidized. Additionally, this potential allows for a fair comparison between the two catalysts, as it is close to the onset of glucose oxidation for both materials. This approach is widely used in electrocatalysis to compare the performance of different catalysts, ensuring that both systems are tested under similar conditions and facilitating a meaningful performance comparison. The time-current graphs demonstrate a stepwise increase in current corresponding to the incremental addition of Glu. The CP/MnO_2_–NRs electrode responds to Glu in 44 s (Figure 6b), while the CNTs/MnO_2_–NRs electrode has a quicker response time of 22 s (Figure 6c). Both electrodes reach steady-state current and show high sensitivity to changes in Glu levels. The CNTs/MnO_2_–NRs electrode’s efficiency stems from to the excellent conductivity and larger surface area of CNTs, enhancing charge transfer and interaction with glucose. Additionally, the unique structural properties of CNTs likely improve electron transport and catalytic activity, contributing to its faster response time. This is supported by the calibration curve showing the sensor’s response current versus Glu concentrations (Figure 6d). Both CP/MnO_2_–NRs and CNTs/MnO_2_–NRs demonstrate two distinct linear relationships across a concentration range from 0.5 mM to 10 mM, well beyond the physiological levels (3–8 mM), with strong correlations indicated by coefficients of determination (R^2^) of 0.9951 for CP/MnO_2_–NRs electrode and 0.9928 for CNTs/MnO_2_–NRs electrode. The sensitivity of CP/MnO_2_–NRs towards Glu, as determined through linear regression (Figure 6d) is estimated to be 176.62 µA cm^−2^ mM^−1^, while the sensitivity of CNTs/MnO_2_–NRs is much higher, at 309.73 µA cm^−2^ mM^−1^. Notably, both electrodes exhibit a similar LOD of 0.19 mM at a signal-to-noise ratio of 3. However, the enhanced sensitivity of the CNTs/MnO_2_–NRs offers a significant advantage, enabling more precise and faster detection of Glu concentrations. While both materials can effectively detect low Glu concentrations, the superior sensitivity of CNTs/MnO_2_–NRs positions them as the preferred choice for sensitive electrochemical sensing applications.

### 3.5. Chronoamperometry for Detection of Interfering Species in Glucose Sensing

**Figure 7:**
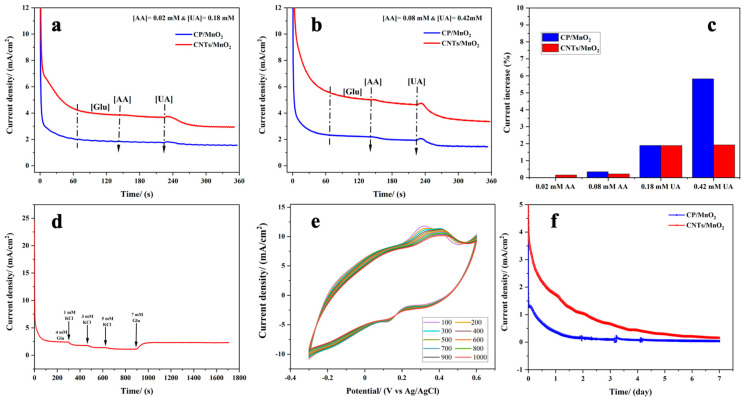
Chronoamperometric (i–t) curve recorded for CP/MnO_2_–NRs and CNTs/MnO_2_–NRs electrodes for 7 mM Glu after addition of: (**a**) AA (0.02 mM) and UA (0.18 mM); (**b**) AA (0.08 mM) and UA (0.42 mM). (**c**) Percentage increase in steady-state current following the addition of UA and AA. (**d**) Chronoamperometric curve after addition of various concentrations of KCl. (**e**) CVs from stability studies upon 1000 cycles at 20 mV/s recorded in 7 mM Glu. The numbers indicate the cycle intervals (every 100 cycles). (**f**) Chronoamperometric long-term durability studies over 7 days in 7 mM Glu.

Oxidizable compounds, UA and AA, commonly co-exist with glucose on physiological fluids and can interfere with glucose detection in various sensors, including non-enzymatic glucose sensors, leading to inaccurate measurements. The typical physiological concentration of Glu, around 3–8 mM, is considerably higher than the concentrations of interfering substances such as UA (0.18–0.42 mM) and AA (0.02–0.08 mM). To evaluate the anti-interference capability of the CP/MnO_2_–NRs and CNTs/MnO_2_–NRs sensors, we focused on both low and elevated concentrations of AA (0.02 mM and 0.08 mM) and UA (0.18 mM and 0.42 mM) that can occur in physiological conditions. Figure 7a,b present comparative interference studies of CP/MnO_2_–NRs and CNTs/MnO_2_–NRs. These studies involved the successive addition of low concentrations of AA (0.02 mM) and UA (0.18 mM), as well as elevated concentrations of AA (0.08 mM) and UA (0.42 mM), to a solution containing 7 mM glucose. Notably, there was no significant change in current densities for either electrode, particularly for the CNTs/MnO_2_–NRs, where the current increase remained below 3% across all interference concentrations tested (Figure 7c). These observations indicate that both the CP/MnO_2_–NRs and CNTs/MnO_2_–NRs electrodes exhibit strong selectivity toward glucose in the presence of potential interferents, such as AA and UA. The minimal change in current densities for the CNTs/MnO_2_–NRs implies a high level of resistance to interference, making it a promising candidate for reliable Glu detection in complex biological samples.

KCl can also potentially foul (or poison) electrodes by adhering to their surfaces, which can compromise sensor performance and selectivity. As a salt, KCl can also alter the ionic strength of the solution, potentially affecting the electrochemical response and stability of the sensor. In physiological blood, KCl concentrations typically range from approximately 3.5 to 5.0 mM [28,29], although individual variations in health and hydration can lead to fluctuations in these levels. Figure 7d shows the current responses of Glu at the CNTs/MnO_2_–NRs upon the successive addition of KCl concentrations of 1 mM, 3 mM, and 5 mM. Remarkably, the current response remains largely unaffected by KCl, even the highest concentration of 5 mM. This resilience underscores the anti-fouling properties of the CNTs/MnO_2_–NRs electrodes, highlighting their effectiveness in maintaining stable sensor performance in the presence of KCl.

The medium-term stability of CNTs/MnO_2_–NRs electrode was first evaluated by CV in the presence of 7 mM Glu, using a scan rate of 20 mV/s over 1000 cycles (equivalent to 25 h). As shown in Figure 7e, the CNTs/MnO_2_–NRs electrode exhibited excellent cyclability, with no significant decrease in current density throughout the duration of the test. To further assess the long-term durability of the electro-catalytic performance of the as-prepared electrodes, oxidation of Glu was conducted with chronoamperometry at a constant voltage of 0.18 V vs. Ag/AgCl. Figure 7f presents a comparison of the current responses of the CP/MnO_2_–NRs electrode and the CNTs/MnO_2_–NRs electrode when exposed to 7 mM glucose in 0.1 M NaOH over a period of 7 days. A chronoamperogram for Glu oxidation typically shows a sharp initial increase in current, indicating rapid oxidation at the electrode surface. This is followed by a sharp decline in current, which is often attributed to the buildup of reaction intermediates, particularly hydrogen peroxide, that progressively block the active sites on the electrode [30,31,32]. After this initial drop, the current stabilizes at a lower, steady-state value, reflecting a dynamic equilibrium between glucose oxidation and the inhibitory effects of the accumulated intermediates. Despite some blockage of active sites, electrochemical activity continues, albeit at a reduced rate, as indicated by the steady-state current. The data shown in Figure 7f highlight the remarkable operational stability of the CNTs/MnO_2_–NRs electrode for amperometric glucose detection. In contrast, the CP/MnO_2_–NRs electrode demonstrated rapid deactivation, likely due to the accumulation of intermediate compounds generated during glucose oxidation, which tended to coat the electrode surface. The superior performance of the CNTs/MnO_2_–NRs electrode can be attributed to its significantly larger electroactive surface area, which not only enhances glucose oxidation efficiency but also helps mitigate surface poisoning by adsorbed intermediates, thus maintaining stable performance over extended periods of operation.

While we acknowledge that NaOH does not replicate physiological conditions (pH~7.4), future studies will focus on testing the sensor in more physiologically relevant solutions (e.g., phosphate-buffered saline) to better mimic the conditions of the human body, ensuring long-term stability and biocompatibility for potential implantable applications.

## 4. Conclusions

In conclusion, the integration of carbon nanotubes (CNTs) with MnO_2_ nanorods into freestanding electrode designs significantly enhances the electrochemical performance of non-enzymatic glucose sensors. The resulting CNTs/MnO_2_ nanorod electrode exhibits a high electrochemical surface area and roughness factor, both of which contribute to increased catalytic sites and improved charge transfer, leading to a high sensitivity of 309.73 µA cm^−2^ mM^−1^ over a broad linear range (0.5–10 mM) and a low detection limit of 0.19 mM, ideal for glucose monitoring in biological systems. The freestanding nature of the electrode provides added advantages, such as better mechanical flexibility, ease of fabrication, and enhanced stability during long-term use, making it highly suitable for real-time glucose detection in complex samples. Additionally, the electrode demonstrates excellent selectivity for glucose, over common interferences (ascorbic acid, uric acid) and exhibits antifouling properties towards KCl. These findings underscore the promising potential of MnO_2_ nanorods–CNTs hybrid structures in advancing the development of cost-effective, high-performance electrochemical sensors for medical and environmental applications.

## Data Availability

Data are contained within the article.
